# Ferulic Acid Protects Against LPS-Induced Sheep Hepatocytes Oxidative Damage via Activating the GSH-GPX4 Pathway and Inhibiting Lipid Metabolism-Mediated Ferroptosis

**DOI:** 10.3390/antiox14101185

**Published:** 2025-09-28

**Authors:** Wenwen Wang, Hongchao Li, Yuan Wang, Na Yin, Jiayu Chen, Yaxuan Niu, Yuchao Hu, Tao Guo, Na Liu, Xiaoping An, Jingwei Qi, Yang Jia, Ruixue Nie

**Affiliations:** 1 College of Animal Science, Inner Mongolia Agricultural University, Hohhot 010018, China; wangwenwen2017@emails.imau.edu.cn (W.W.); lihongchao@emails.imau.edu.cn (H.L.); yinna1997@emails.imau.edu.cn (N.Y.); cjy200204@emails.imau.edu.cn (J.C.); nyx0214@emails.imau.edu.cn (Y.N.); huyuchao@emails.imau.edu.cn (Y.H.); gt199608@emails.imau.edu.cn (T.G.); liuna1988@imau.edu.cn (N.L.); anxiaoping@imau.edu.cn (X.A.); qijingwei@imau.edu.cn (J.Q.); jiayang@imau.edu.cn (Y.J.); nie@imau.edu.cn (R.N.); 2 National Center of Technology Innovation for Dairy-Breeding and Production Research Center, Hohhot 010018, China; 3 Key Laboratory of Smart Animal Husbandry at Universities of Inner Mongolia Autonomous Region, Integrated Research Platform of Smart Animal Husbandry at Universities of Inner Mongolia Autonomous Region, Inner Mongolia Herbivorous Livestock Feed Engineering Technology Research Center, Hohhot 010018, China

**Keywords:** ferulic acid, sheep hepatocytes, ferroptosis

## Abstract

Lipopolysaccharide (LPS) triggers oxidative damage in sheep hepatocytes, linked to ferroptosis. Ferulic acid (FA) is known for its antioxidative properties, but its protective role against LPS via ferroptosis regulation was unclear. The objective of this research is to explore the protective role of FA in mitigating LPS-induced oxidative stress in sheep hepatocytes. The experimental setup consisted of three groups: a control group, an LPS group treated with 10 µg/mL of LPS, and FA group that received both 10 µg/mL of LPS and 750 µg/mL of FA. We found that FA treatment decreased in contents of MDA and LDH. Metabolomics revealed that LPS affected glycerophospholipid metabolism, unsaturated fatty acids biosynthesis, ferroptosis, and arachidonic acid metabolism mainly by reducing the level of PUFAs and LPC in the hepatocyte supernatant, while FA affected glutathione metabolism by increasing L-cysteine, L-ornithine, L-glutamic acid, and L-glutamine. Moreover, transcriptomics demonstrated that the comparison of LPS and control groups were mainly enriched in arachidonic acid metabolism, glycerophospholipid metabolism, and ferroptosis, the comparison of FA and LPS groups was mainly enriched in glutathione metabolism. The results further confirmed the findings in the metabolomics and transcriptomics analyses, showing that LPS treatment upregulated the mRNA expression of *ACSL4*, *LPCAT3*, *ALOX15*, *STEAP3*, *GPX4*, *GCLC*, and *GCL* in hepatocytes, while reducing GSH and GR levels. In contrast, FA intervention attenuated LPS-induced iron overload by decreasing Fe^2+^ accumulation and suppressing the mRNA expression of *ACSL4*, *LPCAT3*, *STEAP3*, and *ALOX15*. Furthermore, FA enhanced the expression of *GPX4*, *GCLC*, *GCLM*, and restored GSH content in LPS-exposed hepatocytes. The above results demonstrated that the protective effect of FA on LPS-induced oxidative damage in the sheep hepatocytes was achieved by activating the GSH-GPX4 pathway and inhibiting lipid metabolism-mediated ferroptosis.

## 1. Introduction

Lambs are frequently fed high-concentrate diets in order to accelerate the fattening process and better align with the increasing market demand for mutton. However, long-term feeding of high-concentrate diets to lambs may cause adverse effects on rumen function, resulting in the massive production of abnormal metabolite lipopolysaccharide (LPS) [1]. LPS originating from the rumen enters the liver via the portal vein, often leading to hepatocytes damage, which reduces the productive performance of lambs and causes significant economic losses to the mutton sheep farming industry [2]. Studies have shown that LPS can induce hepatocyte damage in mice through ferroptosis activation [3,4]. Ferroptosis is an iron-dependent regulated cell death process marked by the buildup of ferrous iron (Fe^2+^) and increased levels of reactive oxygen species (ROS), alongside the peroxidation of polyunsaturated fatty acid (PUFA)-containing phospholipids [5,6]. Notably, rumen-derived LPS could induce ferroptosis in liver tissues in dairy goats [7]. In addition, significant increases in the expression of genes associated with oxidative stress and fatty acid metabolism were observed in the livers of Tibetan sheep fed high-concentrate diets, findings that closely align with the molecular characteristics of ferroptosis [8]. Therefore, ferroptosis may be involved in LPS-induced sheep hepatocytes injury.

Ferulic acid (FA) is an active component of traditional Chinese medicines, possesses various physiological potentials, such as antioxidant, lipid metabolism regulation, anti-apoptotic, and anti-inflammatory [9]. Notably, FA inhibited ferroptosis in the liver of rat by reducing malondialdehyde (MDA) and ROS levels and enhancing expression of glutathione peroxidase 4 (GPX4) [10]. Therefore, FA may become a potential drug for the alleviation of LPS-induced sheep hepatocytes injury, but further studies are lacking to confirm it. Our previous study has found that FA can increase plasma glutathione peroxidase and catalase activities and decrease MDA content of lamb in a cold environment [11]. However, it remains unclear whether FA could protect against LPS-induced liver injury by regulating ferroptosis. The sheep primary hepatocytes have become in vitro model to study protective effect of FA. In this study, we employed multi-omics techniques to explore the role and mechanism of FA in regulating ferroptosis and alleviating liver injury.

## 2. Materials and Methods

### 2.1. Isolation and Culture of Hepatocytes

Lamb primary hepatocytes were obtained from sheep liver tissue using an optimized two-step perfusion technique and cultured following the methodology outlined by Du et al. [12]. Five healthy male lambs (2.9–3.2 kg, 1-day-old, Dorper × Small Tail Han Sheep) from Beichen Co., Ltd. (Baotou, China) were used in this study. Those lambs were intravenously administered heparin to prevent hepatic coagulation and xylazine (0.01 mL/100 kg) for anesthesia. After anesthesia, the collected livers were quickly perfused using solution A (140 mmol/L NaCl, 10 mmol/L HEPES, 6.7 mmol/L KCl, 0.5 mmol/L EDTA, and 2.5 mmol/L glucose, pH 7.4, 37 °C), solution B (140 mmol/L NaCl, 30 mmol/L HEPES, 6.7 mmol/L KCl, 0.5 mmol/L EDTA, 2.5 mmol/L glucose, and 5 mmol/LCaCl_2_, pH 7.4, 37 °C), solution C (0.1 g collagenase IV dissolved in 0.5 L of perfusion solution B) until the perfusion buffer became free of blood. Next 100 mL of fetal bovine serum (Hyclone Laboratories, Logan, UT, USA) was added to stop the digestion, and the liver was cut into pieces with scissors. The cells were filtered using sieves of 100-mesh (150 µm) and 200-mesh (75 µm). Then, they were washed twice in RPMI-1640 basic medium (Hyclone Laboratories), the cell suspension was centrifuged at 1000 rpm for 3 min at 4 °C. Finally, isolated lamb primary hepatocytes were counted and assayed for viability, and were transferred to 6-well plates (1 × 10^6^ cells/mL) or 96-well plates (5 × 10^4^ cells/mL) in the adherent medium (RPMI-1640) supplemented with 10% fetal bovine serum, 10^−6^ M insulin, 10^−6^ M dexamethasone, 10 µg/mL vitamin C, and 1% penicillin/streptomycin under 37 °C in 5% CO_2_. All steps were conducted under sterile conditions.

Lamb primary hepatocytes were seeded in 6-well plates and pre-cultured for 24 h to reach 60–80% confluence. Following pre-culture, cells were exposed to different concentrations of FA (0, 500, 750, 1000 µg/mL) for 12 h. After FA treatment, the medium was carefully aspirated, and the cells were rinsed thoroughly three times using PBS to ensure complete removal of residual medium. Subsequently, cells were subjected to a 12 h treatment under the following conditions. Control group: fresh medium only (no FA, no LPS). LPS group: medium containing 10 µg/mL LPS (L8880, Solarbio, Beijing, China) only. LPS + FA groups: medium containing 10 µg/mL LPS plus the same FA concentrations used in the initial treatment phase (500, 750, or 1000 µg/mL), designated as LPS + FA500, LPS + FA750, and LPS + FA1000, respectively. Samples were collected for subsequent assays. All cell assays were repeated at least three times using independent cells of different batches. FA (purity, 98%) was obtained from Shanghai Yuanye Biotechnology Co., Ltd. (Shanghai, China).

### 2.2. Immunofluorescence

The CK-18 and BSA were measured using the method detailed by Ran et al. [13]. Hepatocytes were washed twice with PBS and fixed with 1 mL of 4% para-formaldehyde per well for 15 min. Then, hepatocytes blocked with solution (0.3% Triton X-100, 5% sheep serum, 94.7% PBS) for 30 min. The cells were incubated overnight at 4 °C with diluted rabbit anti-CK-18 antibody (dilution 1:200; Bioss, bs-1339R) and rabbit anti-BSA antibody (dilution 1:50; Bioss, bs-0292R), continued to incubate with HRP-conjugated goat anti-rabbit IgG H&L (dilution 1:1000; Dakome, S004, Hood River, OR, USA) for 1 h. To visualize the cell nuclei, 1 μg/mL of DAPI was applied for staining. Finally, after drying, the slides were prepared with an anti-fade mounting medium to preserve fluorescence signals, and the resulting images were captured using a fluorescence microscope (Nikon Eclipse C1; Nikon, Tokyo, Japan).

### 2.3. Western Blotting

Western blot analysis was conducted following the previously established protocol [14]. The total protein of hepatocytes was extracted using commercial kit (Thermo Fisher Scientific Inc., Waltham, MA, USA). The protein concentrations of hepatocytes were detected by BCA kit (Applygen, Beijing, China). A total of 60 µg of protein from each sample was separated by SDS-PAGE and transferred to 0.45-µm PVDF membranes. The membranes were first incubated with 3% BSA-TBST at 24 °C for 1 h to block nonspecific binding sites. Following this blocking step, the membranes were incubated overnight at 4 °C with primary antibodies against CK-18 (dilution 1:1000; Bioss, bs-1339R), BSA (dilution 1:500; Bioss, bs-0292R), and β-tubulin (dilution 1:10,000; Immunoway, YM3030). The membranes were washed 3 times and incubated with goat anti-rabbit IgG H&L (dilution 1:10,000; Dakome, S004) secondary antibody at 24 °C for 40 min. The protein bands were developed using enhanced chemiluminescence solution in a protein imager (ProteinSimple, San Jose, CA, USA), then measured using Image J software (Version 1.54, National Institutes of Health, Bethesda, MD, USA).

### 2.4. Cell Viability Assay

Hepatocytes were seeded in 96-well plates at 5 × 10^4^ cells/well and treated with 0, 3, 30, 300, or 3000 µg/mL FA for 24 h or 48 h. For this purpose, 10 μL of the CCK-8 (Solarbio, Beijing, China) were added to each well and the cells were incubated for 3 h at 37 °C later. The optical density was measured by microplate reader (Bio-Rad Laboratories, Hercules, CA, USA) at 450 nm.

### 2.5. Biochemical Assays

The hepatocyte samples (six biological replicates for control, LPS, LPS + FA500, LPS + FA750, and LPS + FA1000 groups) were obtained for the purpose of measuring lactate dehydrogenase (LDH), MDA, total antioxidant capacity (T-AOC), glutathione disulfide (GSSG), glutathione (GSH), superoxide dismutase (SOD), glutathione reductase (GR), total iron (Fe), and Fe^2+^ levels. The LDH, MDA, SOD, T-AOC, GSH, GSSG, GR, Fe, and Fe^2+^ in hepatocytes were measured using the ELISA kits (Nanjing Jiancheng Bioengineering Institute, Nanjing, China) according to the manufacturer’s instructions. Absorbance was read using microplate reader (Bio-Rad Laboratories, USA). The intra-assay coefficients of variation ranged from 3.2% to 5.0%, and inter-assay coefficients of variation ranged from 7.2% to 10.5%.

### 2.6. Untargeted Metabolomics Analysis

The hepatocyte supernatant samples (five biological replicates for control group, six for LPS and LPS + FA750 groups) were sent to Novogene Bioinformatics Technology Co., Ltd. (Beijing, China) for metabolomics analysis. Metabolite extraction and measurements as reported previously with some modifications [15]. Briefly, the supernatant was diluted to achieve a final methanol concentration of 53%. Following dilution, the sample was subjected to centrifugation at 15,000× *g* for 15 min at 4 °C. The supernatant was injected into the LC-MS/MS system for analysis. The original datasets were analyzed with Compound Discoverer 3.3 (CD3.3, Thermo Fisher, Waltham, MA, USA), which was employed to carry out peak alignment, peak detection, and the subsequent quantification of individual metabolites. Variable metabolites were identified by matching the exact masses of the precursor ions and fragment ions to those in online databases such as KEGG (https://www.genome.jp/kegg/pathway.html, accessed on 24 September 2025), HMDB (https://hmdb.ca/metabolites, accessed on 24 September 2025), and LIPIDMaps (http://www.lipidmaps.org/). Principal component analysis (PCA) was performed on sample data using R package Ropls (version 3.21) to analyze differences in metabolic profiles. The volcano plot was generated using the online tools from https://www.bioinformatics.com.cn/. Pathway enrichment analysis was performed using the KEGG database.

### 2.7. Transcriptome Sequencing

Total RNA was extracted from hepatocyte samples (three biological replicates per group for control, LPS, and LPS + FA750 treatments) using TRIzol reagent (Ambion/Invitrogen, Waltham, MA, USA). Following the manufacturer’s protocol, the sequencing libraries were sequenced on Illumina NovaSeq 6000 platform by Novogene Bioinformatics Technology Co., Ltd. (Beijing, China). Raw reads underwent quality assessment and filtering to eliminate adapter contamination and low-quality sequences, a process managed using custom perl scripts developed in-house. The resulting high-quality, cleaned reads were subsequently analyzed to determine key quality metrics, including the proportions of bases with Q20 and Q30 quality scores as well as the overall GC content. The quality-controlled clean reads aligned to the reference genome by ARS-UI_Ramb_v3.0 using HISAT2. The quantification of transcript levels was performed using the RSEM tool, with subsequent normalization employing the FPKM approach to standardize expression values across samples. DEGs annotation information and enrichment pathways were analyzed using the KEGG database. The volcano plot was generated using the online tools from https://www.bioinformatics.com.cn/.

### 2.8. Quantitative Real-Time PCR Analysis

Total RNA was extracted from hepatocyte samples (six biological replicates each for control, LPS, and LPS + FA750 groups) using TRIzol reagent (Invitrogen, Waltham, MA, USA), as described previously [16]. Prior to cDNA synthesis, RNA purity and integrity were, respectively, assessed by OD260/OD280 ratios (ranging from 1.8 to 2.0 across all samples) and electrophoresis on 1% agarose gels. According to the manufacturer’s instructions, 1 μg of total RNA was utilized to synthesize cDNA in a 20 μL reaction mixture. The conditions for qRT-PCR System (Roche Diagnostics, Basel, Switzerland) were as follows: 95 °C for 1 min for cycle, followed by 40 cycles of 95 °C for 15 s and 63 °C for 25 s. The mRNA expression of the target genes was normalized using β-actin as the reference gene. The quantification was performed employing the 2^−ΔΔCt^ method to determine relative expression values. The primer sequences for *GPX4*, glutamate cysteine ligase catalytic subunit (*GCLC*), glutamate cysteine ligase modifier subunit (*GCLM*), cysteine–glutamate ligase (*GCL*), Acyl-CoA synthetase long-chain family member 4 (*ACSL4*), lysophosphatidylcholine acyltransferase 3 (*LPCAT3*), arachidonate 15-lipoxygenase (*ALOX15*), six-transmembrane epithelial antigen of prostate 3 (*STEAP3*), and *β-actin* are listed in Table 1.

### 2.9. Statistical Analysis

The phenotypic data were conducted using SAS 9.2 (SAS Institute, Cary, NC, USA), with an initial assessment of variance homogeneity performed prior to executing the main analytical procedures. The lamb primary hepatocytes were seeded in 6-well plates serving as the experimental unit. The two-tailed unpaired Student’s t-test was conducted to determine differences between groups. The results were expressed as means and SD. Values *p* < 0.05 were taken to indicate the significance. The metabolites with VIP > 1, *p*-value < 0.05, and fold change ≥ 1.2 or fold change ≤ 0.833 were considered to be differential metabolites. Differentially expressed genes (DEGs) analysis was performed using DESeq2. When |log2foldchange| ≥ 1 and *p* < 0.05, genes were considered DEGs.

## 3. Results

### 3.1. Identification of Hepatocytes

**Figure 1:**
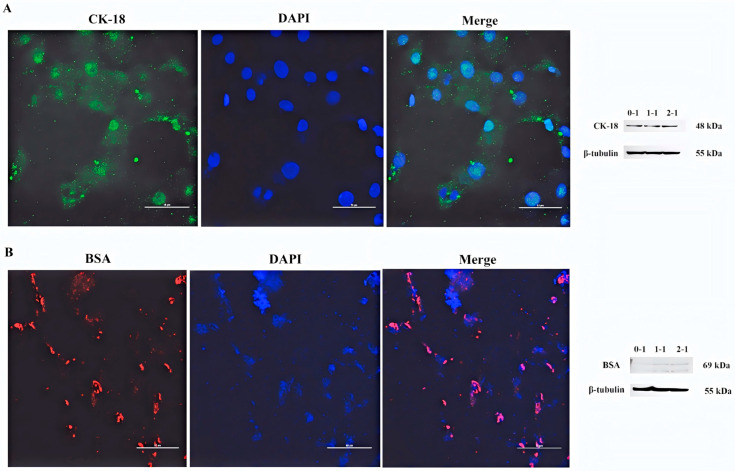
Identification of CK-18 and BSA by immunofluorescence and Western blot in sheep primary hepatocytes. (**A**) Immunofluorescence and Western blot were used to detect the protein expression of CK-18 in the cells. Using DAPI to stain the nucleus is blue, and staining CK-18 by fluorescent secondary antibody FITC coupling is green. (**B**) Immunofluorescence and Western blot were used to detect the protein expression of BSA in the cells. Using DAPI to stain the nucleus is blue, and staining BSA by fluorescent secondary antibody FITC coupling is red. Scale = 50μm.

CK-18 and BSA serve as highly specific markers that are predominantly expressed in normal liver parenchymal cells, making them reliable indicators for the identification and characterization of hepatocytes. By immunofluorescence experiments, most of the cells had specific green fluorescence and red fluorescence, indicating that the cells were positive for CK-18 and BSA (Figure 1). Moreover, Western Blot analysis revealed that CK-18 and BSA expression were observed across the samples (Figure 1).

### 3.2. Ferulic Acid Ameliorated LPS-Induced Sheep Hepatocytes Oxidative Damage

Following FA treatment, the cell viability was not affected (*p* > 0.05) when the concentration of FA was <3000 μg/mL for 24 h (Figure S1). However, when concentrations were over 300 μg/mL for 48 h, cell viability decreased compared with control group (*p* < 0.05). Therefore, the optimal incubation time for FA is 24 h, and the suitable dosage range is 0–3000 μg/mL.

**Figure 2:**
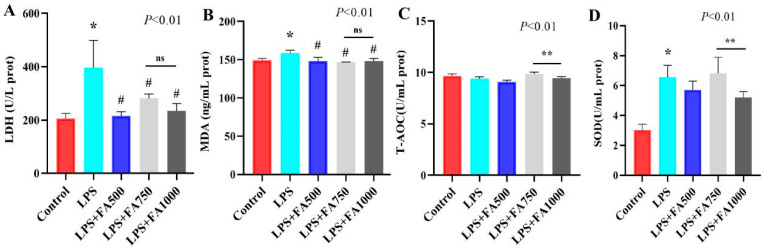
Ferulic acid ameliorated LPS-induced sheep hepatocytes oxidative damage. (**A**) The LDH content in the sheep hepatocytes from each group. (**B**) The MDA content in the sheep hepatocytes from each group. (**C**) The T-AOC level in the sheep hepatocytes from each group. (**D**) The SOD content in the sheep hepatocytes from each group. The data were represented as mean ± SD. Statistical significance is indicated by * *p* < 0.01, (Control vs. LPS); # *p* < 0.01, (LPS + FA500, LPS + FA750, LPS + FA1000 vs. LPS); or ** *p* < 0.01, (LPS + FA750 vs. LPS + FA1000). ns, not significant. Control: control group; LPS: LPS group; LPS + FA500: LPS + FA500 group; LPS + FA750: LPS + FA750 group; LPS + FA1000: LPS + FA1000 group.

We established an oxidative damage model with LPS in sheep hepatocytes. As shown in Figure 2, the content of LDH, MDA, and SOD increased (*p* < 0.01) compared with control group. FA treatment reduced LDH and MDA content compared to the LPS group (*p* < 0.01). Notably, the concentrations of T-AOC and SOD was higher (*p* < 0.01) for LPS + FA750 group than LPS + FA1000 group.

### 3.3. Ferulic Acid Extensively Modulated Metabolomic Profile of Sheep Hepatocytes with LPS

**Figure 3:**
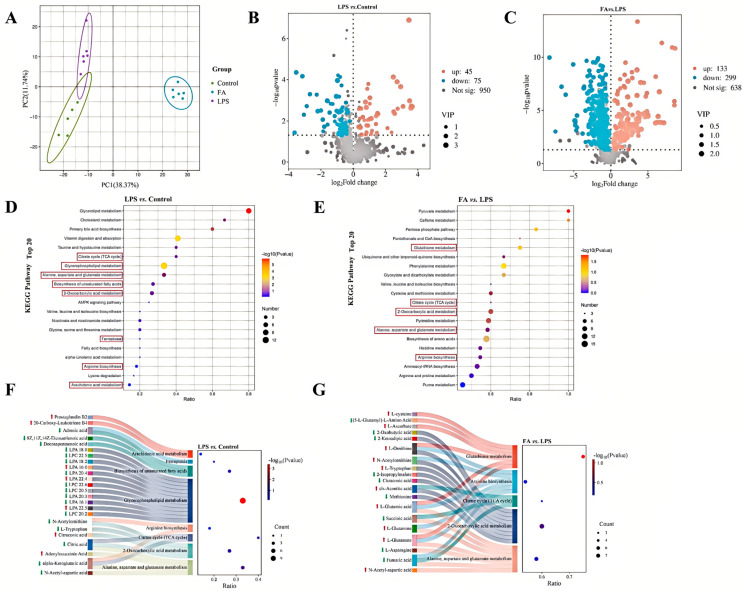
Ferulic acid extensively modulated metabolomic profile of sheep hepatocytes with LPS. (**A**) Principal component analysis score plot. (**B**) The volcano plot displayed all the metabolites identified in both the LPS and the control group. (**C**) The volcano plot showed all metabolites detected from the FA and LPS groups. (**D**) Top 20 KEGG pathways of differential metabolites between the LPS and control groups. (**E**) Top 20 KEGG pathways of differential metabolites between the FA and LPS groups. (**F**) The differential metabolites enriched in main pathways between the LPS and control groups. (**G**) The differential metabolites enriched in main pathways between the FA and LPS groups. Control: control group; LPS: LPS group; FA: LPS + FA750 group.

To validate FA’s protective effects against LPS-induced oxidative damage in hepatocytes, we conducted untargeted metabolomics to analyze supernatant metabolite profiles across control, LPS, and LPS + FA750 treatment groups. PCA showed the three groups were separated, revealing different metabolomic profiles among groups (Figure 3A). Compared to the control group, the LPS group exhibited a total of 120 differential metabolites, with 45 upregulated and 75 downregulated (Figure 3B). Between the FA and LPS groups, 432 differential metabolites were identified, with 133 upregulated and 299 downregulated (Figure 3C). The differential metabolites were subjected to pathway analysis based on the KEGG database. The top 20 pathways were selected for the compare of control and LPS groups, and the main pathways included glycerophospholipid metabolism, biosynthesis of unsaturated fatty acids, ferroptosis, and arachidonic acid metabolism (Figure 3D). The top 20 pathways were selected for the compare of FA and LPS groups, and the main pathway included glutathione metabolism (Figure 3E). Additionally, KEGG enrichment analysis revealed that the two comparison groups were mainly enriched in the alanine, aspartate and glutamate metabolism, 2-Oxocarboxylic acid metabolism, citrate cycle (TCA cycle), and arginine biosynthesis pathway. Therefore, we selected differential metabolites enriched in glycerophospholipid metabolism, biosynthesis of unsaturated fatty acids, ferroptosis, arachidonic acid metabolism, alanine, aspartate and glutamate metabolism, 2-Oxocarboxylic acid metabolism, citrate cycle (TCA cycle), and arginine biosynthesis pathways and demonstrated their distribution between the control and LPS groups (Figure 3F). Compared to the control group, the levels of 20-Carboxy-Leukotriene B4, Prostaglandin B2, LPA 16:0, LPA 22:4, LPA 22:5, citraconic acid, and adenylocuccinic acid were increased (*p* < 0.05), while the levels of adrenic acid, docosapentaenoic acid, 8Z,11Z,14Z-Eicosatrienoic acid, LPA 18:0, LPC 20:5, LPA 18:2, LPA 20:4, LPC 22:6, LPA 20:3, LPA 16:1, LPC 20:2, LPC 22:5, N-Acetylornithine, L-Tryptophan, alpha-Ketoglutaric acid, citric acid, and N-Acetyl-aspartic acid were decreased (*p* < 0.05) in the LPS group (Figure 3F). We selected differential metabolites enriched in glutathione metabolism, alanine, aspartate and glutamate metabolism, 2-Oxocarboxylic acid metabolism, citrate cycle (TCA cycle), and arginine biosynthesis pathways and demonstrated their distribution between the FA and LPS groups (Figure 3G). The level of L-cysteine, L-Ascorbate, L-Ornithine, N-Acetylornithine, L-Tryptophan, cis-Aconitic acid, L-Glutamic acid, L-Glutamine, L-Glutamate, and N-Acetyl-aspartic acid had a increase (*p* < 0.05) in the FA group compared with the LPS group, while the level of (5-L-Glutamyl)-L-Amino Acid, 2-Ketoadipic acid, 2-Oxobutyric acid, 2-Isopropylmalate, citraconic acid, methionine, succinic acid, L-Asparagine, and fumaric acid had a decrease (*p* < 0.05).

### 3.4. Ferulic Acid Extensively Regulated the Expression of Genes Involved in Ferroptosis

**Figure 4:**
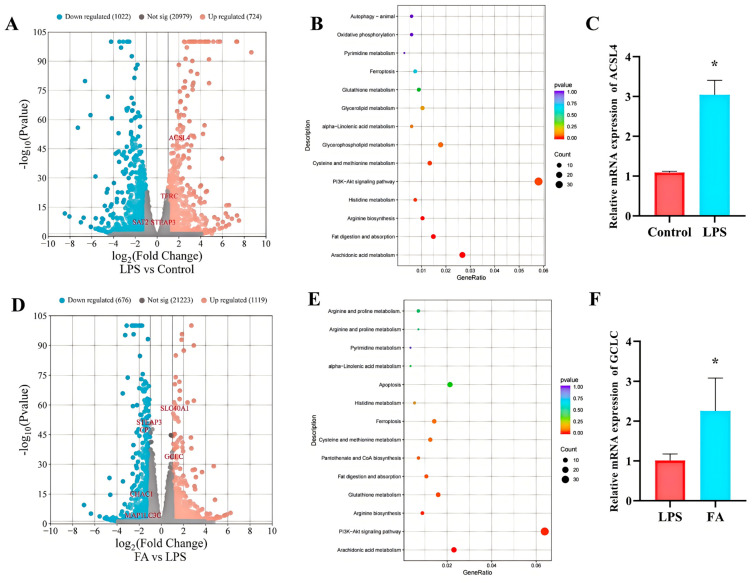
Ferulic acid extensively regulated the expression of genes involved in ferroptosis. (**A**) The volcano plot showed all genes detected from the LPS and control groups. (**B**) KEGG pathways of differential genes between the LPS and control groups. (**C**) The relative mRNA levels of ACSL4 were determined by real-time PCR. (**D**) The volcano plot showed all genes detected from the FA and LPS groups. (**E**) KEGG pathways of differential genes between the FA and LPS groups. (**F**) The relative mRNA levels of GCLC were determined by real-time PCR. Control: control group; LPS: LPS group; FA: LPS + FA750 group. Statistical significance is indicated by * *p* < 0.01.

RNA-seq-based transcriptomic analysis revealed the underlying mechanisms and potential molecular targets through which LPS and FA regulate ferroptosis. This generated 58.93 GB of raw data from nine libraries, with ≥93.89% of reads at Q30 quality or higher (Table S1). In the annotation files for sequences that aligned uniquely, 91.70%, 6.12%, and 2.18% of reads aligned to exon, intron, and intergenic regions, respectively, per sample (Figure S2). A total of 1746 genes were identified as differential expression gene (DEGs, *p*-value < 0.05 and |log2FC| ≥ 1) in the LPS group when compared to control group, with 724 upregulated and 1022 downregulated. A total of 1795 genes were identified as differential expression gene (DEGs, *p*-value < 0.05 and |log2FC| ≥ 1) in the FA group when compared to LPS group, with 1119 upregulated and 676 downregulated. KEGG pathway enrichment analysis indicated that altered genes of control and LPS groups were enriched in pathways, such as arachidonic acid metabolism, fat digestion and absorption, arginine biosynthesis, histidine metabolism, PI3K-Akt signaling pathway, cysteine and methionine metabolism, glycerophospholipid metabolism, alpha-Linolenic acid metabolism, glycerolipid metabolism, glutathione metabolism, ferroptosis, pyrimidine metabolism, oxidative phosphorylation, and autophagy—animal (Figure 4B). The differential expression gene of FA and LPS groups were enriched in pathways, such as arachidonic acid metabolism, PI3K-Akt signaling pathway, arginine biosynthesis, glutathione metabolism, fat digestion and absorption, pantothenate and CoA biosynthesis, cysteine and methionine metabolism, ferroptosis, histidine metabolism, apoptosis, alpha-Linolenic acid metabolism, pyrimidine metabolism, arginine and proline metabolism, and oxidative phosphorylation (Figure 4E). Through real-time PCR validation of potential target genes (ACSL4 and GCLC) selected from differential gene KEGG enrichment analysis, ACSL4 mRNA expression was further confirmed to be upregulated after LPS treatment (Figure 4C). Moreover, GCLC is the potential target gene for FA in alleviating ferroptosis in sheep hepatocytes with LPS (Figure 4F).

### 3.5. Ferulic Acid Protects Against LPS-Induced Sheep Hepatocytes Injury via Regulating Lipid Metabolism-Mediated Ferroptosis

**Figure 5:**
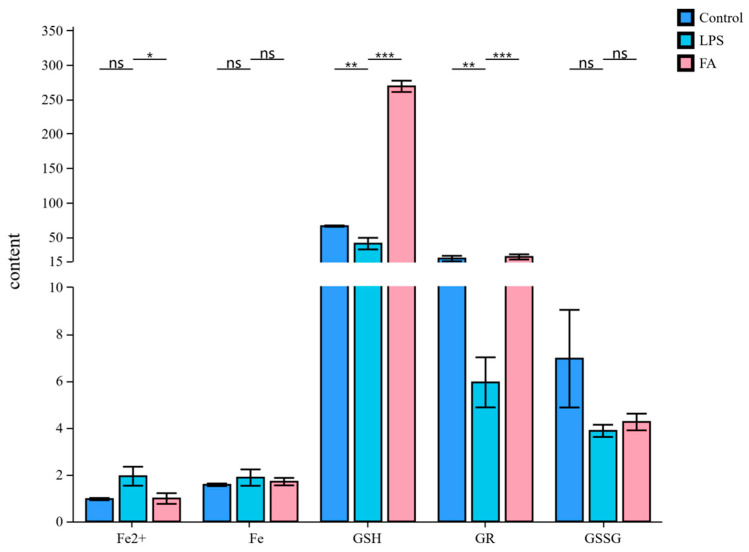
Bar plot of Fe^2+^, Fe, GSH, GR, and GSSG contents in the sheep hepatocytes. Data are presented as mean ± SD. * *p* < 0.05, ** *p* < 0.01, *** *p* < 0.001; ns, not significant. Control: control group; LPS: LPS group; FA: LPS + FA750 group.

**Figure 6:**
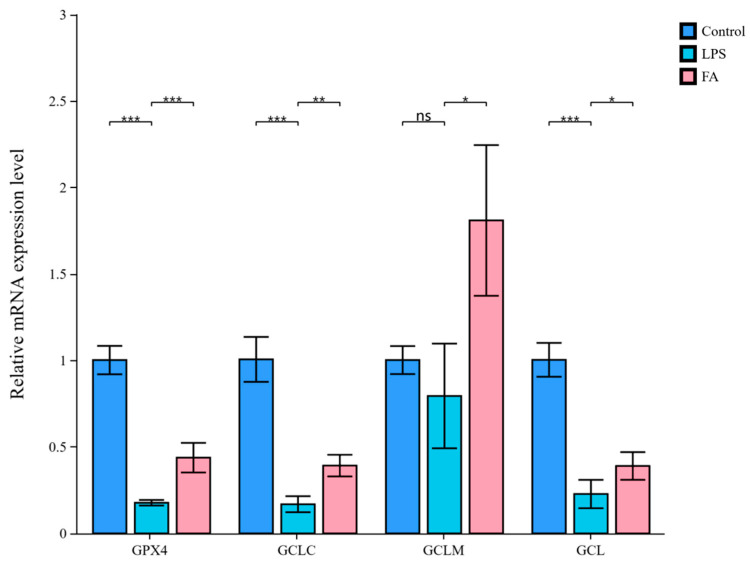
Bar plot of GPX4, GCLC, GCLM, and GCL gene expression in the sheep hepatocytes. Data are presented as mean ± SD. * *p* < 0.05, ** *p* < 0.01, *** *p* < 0.001; ns, not significant. Control: control group; LPS: LPS group; FA: LPS + FA750 group.

**Figure 7:**
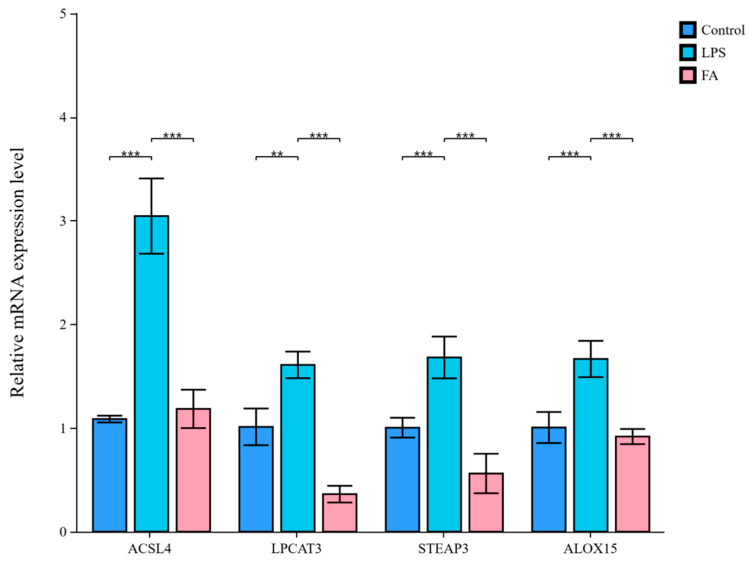
Bar plot of ACSL4, LPCAT3, STEAP3, and ALOX15 gene expression in the sheep hepatocytes. Data are presented as mean ± SD. ** *p* < 0.01, *** *p* < 0.001. Control: control group; LPS: LPS group; FA: LPS + FA750 group.

Figure 5 shows that the contents of GSH and GR in the LPS group were decreased compared with the control group (*p* < 0.01), whereas a increase was observed in the FA group (*p* < 0.001). Notably, FA treatment reduced Fe^2+^ levels and enhanced GSH contents in the sheep hepatocytes with LPS (*p* < 0.05). There was no difference between the LPS and FA group regarding the contents of Fe and GSSG. As shown in Figure 6, compared with the control group, the relative mRNA expression levels of GPX4, GCLC, and GCL in the LPS group were decreased (*p* < 0.001). In contrast, GPX4, GCLC, GCLM, and GCL gene expression in the FA group was higher than in the LPS group (*p* < 0.05). In addition, the gene expression of ACSL4, LPCAT3, STEAP3, and ALOX15 were upregulated by LPS when compared to control group (*p* < 0.01, Figure 7). However, the relative mRNA expression of ACSL4, LPCAT3, STEAP3, and ALOX15 in the FA group were lower than in the LPS group (*p* < 0.001, Figure 7).

## 4. Discussion

### 4.1. FA Protects Against LPS-Induced Sheep Hepatocytes Oxidative Damage

LPS originating from the rumen causes liver oxidative damage and seriously impacts sheep health. Some studies have confirmed that FA has a strong ability to alleviate oxidative stress [17,18]. Here, we utilized sheep primary hepatocytes as a model to investigate the underlying mechanisms of how FA might exert protective effects against LPS-induced liver injury. LDH is a potential marker of cell injury [19]. MDA as a biomarker of oxidative stress [20]. In the present study, the level of LDH, MDA, and SOD were enhanced after LPS treatment, demonstrating hepatocytes oxidative damage. However, FA treatment significantly reduced LDH and MDA content, confirming that FA is capable of ameliorating LPS-induced sheep hepatocytes oxidative damage. Our results are consistent with Shi et al. [21], who also reported FA pretreatment can protect hepatocytes from oxidative damage by decreasing LDH and MDA levels. The high SOD concentration in LPS group may be responsible for the stimulation of LPS-induced oxidative damage. Previous research has demonstrated that after 24 h of LPS treatment, macrophages derived from LPS-sensitive and LPS-resistant mice exhibited elevated levels of MnSOD mRNA and protein [22]. Note that the level of T-AOC and SOD was higher for the LPS + FA750 group than LPS + FA1000 group. Similarly to the findings of our study, moderate concentrations of FA (0.25~2 mmol/L) were shown to reduce ethanol-induced p-H2AX expression and decrease ROS levels in LO2 cells. In contrast, high concentrations of FA (8 mmol/L) demonstrated toxic effects on the cells [23]. Therefore, supplementation with 750 μg/mL is suitable for improving antioxidative capability of hepatocytes. The untargeted metabolomics and transcriptome sequencing of hepatocytes in the control, LPS and LPS + FA750 (FA group) groups were analyzed. Subsequently, the mechanisms underlying hepatocytes oxidative damage caused by LPS and the protective effect of FA were further elucidated, during which several potential biomarkers were identified.

### 4.2. LPS Triggers Lipid Metabolism-Mediated Ferroptosis in Hepatocytes

Untargeted metabolomics results demonstrated that LPS treatment primarily disrupts glycerophospholipid metabolism, unsaturated fatty acid biosynthesis, ferroptosis, and arachidonic acid metabolism in hepatocytes by reducing the supernatant levels of polyunsaturated fatty acids (PUFAs)-specifically adrenic acid, docosapentaenoic acid, and 8Z,11Z,14Z-eicosatrienoic acid, and lysophosphatidylcholines (LPCs), including LPC 20:5, LPC 22:6, LPC 20:2, and LPC 22:5. Notably, adrenic acid, docosahexaenoic acid, and LPCs are raw materials for the formation of PUFA-phospholipids [24]. However, the peroxidation of PUFA-phospholipids in membranes is a classic mechanism of ferroptosis [25]. The peroxidation of PUFA-phospholipids is primarily divided into the following three stages. The first step involves the activation of PUFAs, catalyzed by ACSL4, which forms PUFA-CoA [26]. The second step is their incorporation into membranes: PUFA-CoA is esterified to lysophosphatidylethanolamine (LPE) or LPC by lysophosphatidylcholine acyltransferase 3 (LPCAT3), generating PUFA-PE or PUFA-PC, which are subsequently integrated into cellular membranes [27]. The third step requires peroxidation, mediated by arachidonate lipoxygenases (ALOXs), which produces lipid hydroperoxides (PUFA-PLOOH) and, secondarily, aldehyde products such as MDA [28]. Zhang et al. reported that LPS treatment upregulated the mRNA and protein expression of ACSL4, and increased MDA and cellular Fe^2+^ content in bovine hepatocytes [29]. Thus, our findings suggest that the depletion of LPCs and PUFAs in the hepatocyte culture supernatant following LPS treatment is likely attributable to their intracellular consumption during ferroptosis induction, as evidenced by enhanced MDA content. Meanwhile, transcriptome sequencing found that ferroptosis was identified between the control and LPS groups. Real-time PCR results further confirmed the findings in untargeted metabolomics and transcriptomic analysis, showing that increased ACSL4, LPCAT3, ALOX15, and STEAP3 mRNA expression in the hepatocyte indicate the presence of ferroptosis in the LPS group. STEAP3 was previously proved to serve as a key regulator in ferroptosis via reducing Fe^3+^ to Fe^2+^ [30]. Our findings indicated that the level of Fe^2+^ increased by one-fold after LPS treatment. Based on the present results and references, we speculated that LPS triggers lipid metabolism-mediated ferroptosis in hepatocytes, consistent with previous study [3].

### 4.3. FA Inhibits Lipid Metabolism-Mediated Ferroptosis by Modulating the GSH-GPX4 System

In this study, KEGG enrichment analysis of untargeted metabolomics and transcriptomics revealed that the comparison of FA and LPS groups were mainly enriched in glutathione metabolism. Consistent with our observations, a recent study demonstrated that puerarin significantly suppresses inflammatory responses in LPS-induced RAW264.7 macrophages by modulating the ferroptosis-associated glutathione metabolism pathway, as revealed through integrated network pharmacology and metabolomics analyses [31]. Furthermore, the level of L-Cysteine, L-Ornithine, N-Acetylornithine, L-Glutamic acid, L-Glutamine, L-Glutamate, and N-Acetyl-aspartic acid had a significant increase in the FA group compared with the LPS group. Cysteine and glutamate are raw materials for the synthesis of GSH [32]. These increased characteristic metabolites in the hepatocyte supernatant may originate from the catabolism of GSH, suggesting a significant improvement in GSH biosynthesis given FA supplement. To test the hypothesis, we quantified cellular GSH levels, and the mRNA expression of GCL, GCLC, and GCLM. The first and rate-limiting step of GSH biosynthesis is catalyzed by GCL, which was composed of GCLC and modifier GCLM subunits [33]. Biochemical assays found that the contents of GSH in the LPS group were decreased compared with the control group, whereas a significant increase was observed in the FA group. Similarly to our findings, Wang et al. [34] also discovered that treatment with FA to PC12 cell increased GSH levels. Current study revealed that FA upregulated the mRNA expression levels of GCLC and GCLM in the sheep hepatocyte, consistent with a previous study [35]. Overall, these findings support the conclusion that FA could increase GSH biosynthesis.

**Figure 8:**
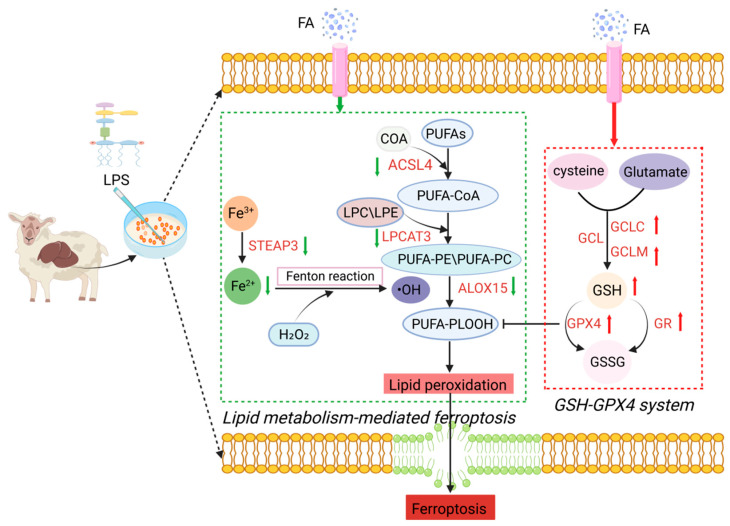
The mechanism of FA protecting against LPS-induced sheep hepatocytes oxidative damage via activating the GSH-GPX4 pathway and inhibiting lipid metabolism-mediated ferroptosis. FA: ferulic acid; LPS: lipopolysaccharide; STEAP3: six-transmembrane epithelial antigen of prostate 3; ACSL4: acyl-CoA synthetase long-chain family member 4; LPCAT3: lysophosphatidylcholine acyltransferase 3; ALOX15: arachidonate 15-lipoxygenase; GCLC: glutamate cysteine ligase catalytic subunit; GCLM: glutamate cysteine ligase modifier subunit; GCL: cysteine–glutamate ligase; GSH: glutathione; GSSG: glutathione disulfide; GR: glutathione reductase; GPX4: glutathione peroxidase 4; PUFAs: polyunsaturated fatty acids; LPE/LPC: lysophosphatidylethanolamine/lysophosphocholine; PLOOH: phospholipid hydroperoxides; PE/PC: phosphatidylethanolamine/phosphocholine.

The GSH-GPX4 system is known to suppress lipid metabolism-mediated ferroptosis [36]. The deprivation of intracellular GSH and the deactivation of GPX4 impede the elimination of lipid peroxides generated through the Fe^2+^-catalyzed Fenton reaction via GPX4-mediated reduction [37]. When cells are exposed to oxidative stress, GR catalyzes the conversion of GSSG to GSH, providing reducing power for the elimination of ROS and other oxidants, thereby maintaining intracellular redox homeostasis and resisting intracellular oxidative stress [38]. Our findings showed that FA treatment enhanced GR activity and GPX4 mRNA expression compared with the LPS group. Similarly, a previous study has demonstrated that FA treatment could salvage the LPS-induced reduction in GPX4 of MLE-12 cells [18]. In the present study, FA treatment significantly suppressed expression of ferroptosis mediators (ACSL4, LPCAT3, ALOX15, STEAP3) and decreased intracellular Fe^2+^ levels in hepatocytes. FA is a hydroxycinnamic acid belonging to the phenolic compound class, could modulate ferroptosis [39]. This observed iron-regulatory effect aligns with findings for other natural compounds. Specifically, acacetin upregulated GPX4 expression while downregulating ACSL4 in HepG2 cells [40]. Similarly, anhydroxysafflor yellow B reduced Fe^2+^ levels in PC12 cells [41]. Furthermore, baicalein decreased iron accumulation, inhibited ALOX15 expression, and reduced lipid peroxidation in CPT-11-induced gastrointestinal dysfunction in Wistar rats [42]. Taken together, these findings demonstrate that FA suppresses lipid metabolism-mediated ferroptosis in hepatocytes through three interconnected mechanisms: (1) enhancing GSH biosynthesis; (2) upregulating GPX4 expression; (3) inhibiting key lipid peroxidation regulators (including ACSL4, LPCAT3, ALOX15, and STEAP3). Collectively, this action potentiates cellular antioxidant capacity and mitigates LPS-induced hepatic injury. The probable mechanism by which FA counters oxidative stress is shown in Figure 8.

## 5. Conclusions

In summary, LPS treatment resulted in Fe^2+^ accumulation, increased lipid peroxidation, and ultimately triggered lipid metabolism-mediated ferroptosis in hepatocytes. Our research underscored that FA inhibit lipid metabolism-mediated ferroptosis through enhancing the GSH-GPX4 antioxidant system, potentially by promoting GPX4 expression and GSH biosynthesis or suppressing ACSL4, LPCAT3, ALOX15, STEAP3 expression.

## Figures and Tables

**Table 1:** Primer sequence used for quantitative qRT-PCR.

Gene	Nucleotide Sequences 5′ to 3′	Size/bp
*GPX4*	F:GAGTTCGCTGCTGGCTAT R:CTTGGGCTGGACTTTCAT	105
*GCLC*	F:CTGGATGATGCCAACGAGT R:CCACGAACACCACATACGC	173
*GCLM*	F:ACGGGGAACCTGCTGAA R:CTGGGCTGATTTGGGAAC	118
*GCL*	F:GAACAAGACAGTGAGGTGGGG R:AGCAGGTCAAAGCCGAAG	140
*ACSL4*	F:GCCCACCTCAGACAAAC R:TATTCACTCTGCGGTTC	263
*LPCAT3*	F:CGCTGGCTTCTCCTACT R:ATGGTGCTGTTTGGTATCT	123
*STEAP3*	F:TGGCTGGGCTGTTTCCT R:TGCTCCTGCTCTGTGGG	175
*ALOX15*	F:CAAGGCTGTGCTGAAGAA R:TGGTTGGTGGAAGAGGG	176

## Data Availability

The datasets during the current study are available from the corresponding author on reasonable request. The transcriptome data have been submitted to SRA (PRJNA1224196). The SRA metadata are available at https://dataview.ncbi.nlm.nih.gov/object/PRJNA1224196?reviewer=vilc5f58kbh7nbcv2ntl2dt5tu in read-only format (accessed on 15 February 2025).

## Supplementary Materials

The following supporting information can be downloaded at https://www.mdpi.com/article/10.3390/antiox14101185/s1. Figure S1: The effects of ferulic acid on cell viability for 24 h and 48 h; Figure S2: Statistical diagram for the distribution of reads mapped to the reference genome; Table S1: Summary of sequencing quality dataset.

## Abbreviations

The following abbreviations are used in this manuscript:

LPS	Lipopolysaccharide
Fe^2+^	Iron(II) ion
ROS	Reactive oxygen species
PUFA	Polyunsaturated fatty acid
FA	Ferulic acid
MDA	Malondialdehyde
GPX4	Glutathione peroxidase 4
PBS	Phosphate-buffered saline
SOD	Superoxide dismutase
T-AOC	Total antioxidant capacity
GSH	Glutathione
GSSG	Glutathione disulfide
GR	Glutathione reductase
LDH	Lactate dehydrogenase
GCLC	Glutamate cysteine ligase catalytic subunit
GCLM	Glutamate cysteine ligase modifier subunit
GCL	Cysteine–glutamate ligase
ACSL4	Acyl-CoA synthetase long-chain family member 4
LPCAT3	Lysophosphatidylcholine acyltransferase 3
ALOX15	Arachidonate 15-lipoxygenase
STEAP3	Six-transmembrane epithelial antigen of prostate 3
