# Supplementary material for: Ferulic Acid Protects Against LPS-Induced Sheep Hepatocytes Oxidative Damage via Activating the GSH-GPX4 Pathway and Inhibiting Lipid Metabolism-Mediated Ferroptosis
Source: Antioxidants (Basel). 2025 Sep 28;14(10):1185. doi: 10.3390/antiox14101185 (PMC12562129; doi:10.3390/antiox14101185)
Supplement: Supplementary file 1 [file antioxidants-14-01185-s001.zip › antioxidants-3870729-supplementary.pdf]

Table S1. Summary of sequencing quality dataset.

Sample [1]	Raw Reads	Raw Bases (G)	Clean Reads	Clean Bases (G)	Q20 [2] (%)	Q30 [2] (%)	GC content (%)
Control_1	46632650	6.99G	46527564	6.98G	97.59	93.45	53.06
Control_2	40547544	6.08G	38040650	5.71G	97.78	93.75	52.77
Control_3	48443230	7.27G	46433942	6.97G	97.8	93.79	51.99
LPS_1	42143228	6.32G	40234256	6.04G	97.94	94.19	52.08
LPS_2	40660064	6.1G	39083706	5.86G	97.93	94.2	52.46
LPS_3	40924356	6.14G	38917986	5.84G	97.87	94.06	52.69
FA_1	39577106	5.94G	37864760	5.68G	97.93	94.16	52
FA_2	46819646	7.02G	44431500	6.66G	97.77	93.69	52.03
FA_3	47105536	7.07G	45402014	6.81G	97.8	93.74	51.51

[1] 9 samples from control, LPS, and FA groups. [2] represents the proportion of 1 incorrectly identified base per 1000 bases, with a correct recognition rate of 99.9%.

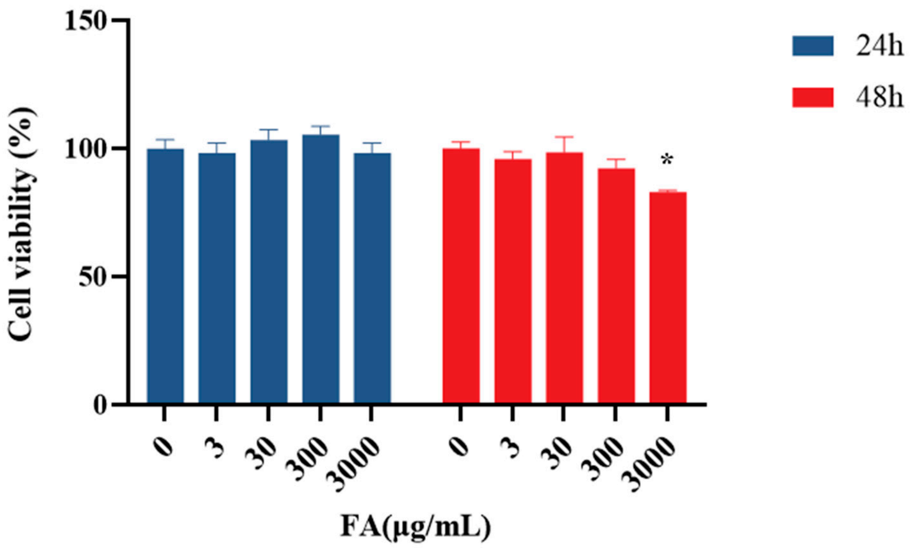

Figure S1. The effects of ferulic acid on cell viability for 24 h and 48 h. Data are presented as mean ± SD. * $p < 0.05$. FA: ferulic acid.

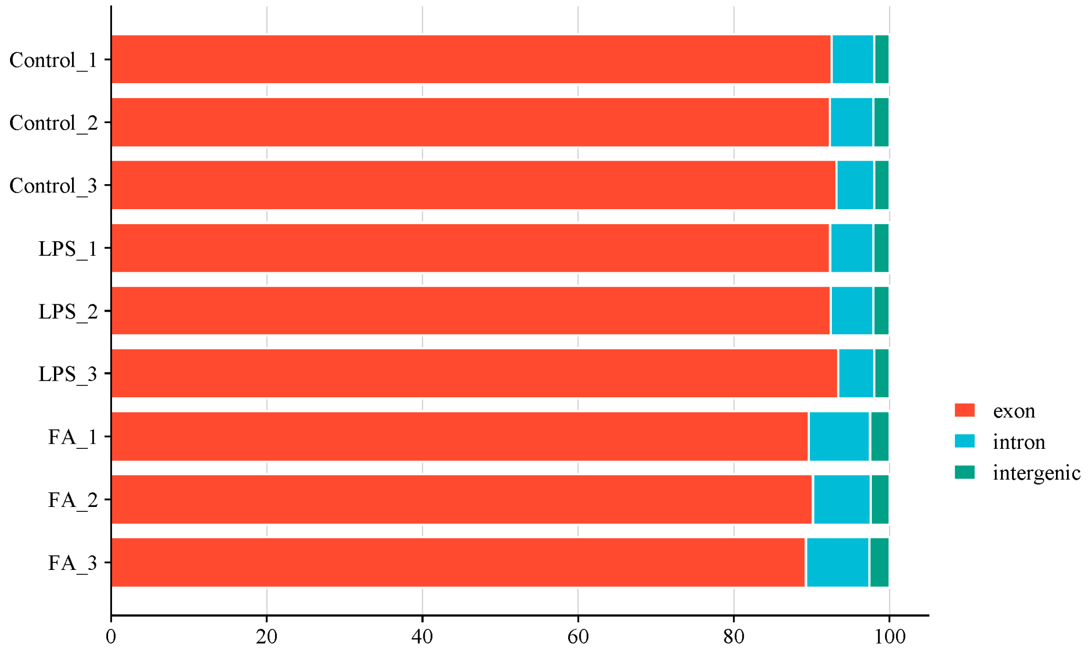

Figure S2. Statistical diagram for the distribution of reads mapped to the reference genome.
